# Development and validation of an interpretable machine learning model for predicting chronic atrophic gastritis in elderly patients

**DOI:** 10.3389/fmed.2026.1810290

**Published:** 2026-07-02

**Authors:** Wenjing Fan, Jinyu Wang, Lu Li, Chao Tian, Zhiwei Yang, Guangchao Zhang, Deyu Xu, Xingtang Yang

**Affiliations:** 1 Shanghai Tenth People’s Hospital, School of Medicine, Tongji University, Shanghai, China; 2 School of Medicine, Tongji University, Shanghai, China

**Keywords:** chronic atrophic gastritis, elderly patients, machine learning, risk factor, SHAP

## Abstract

**Background:**

Chronic atrophic gastritis (CAG) is a key precancerous condition, but its screening in the elderly is hindered by the invasiveness, high cost, and limited accessibility of endoscopy. This study aimed to develop and validate interpretable machine learning (ML) models for predicting CAG in elderly patients and identify key predictive factors.

**Methods:**

We adopted a retrospective cohort study design using data from the Endoscopy Department of Shanghai Tenth People’s Hospital. Two independent cohorts were included: a development cohort (January 2023 to December 2024, *n* = 1,268) for model construction and internal validation, and a temporal validation cohort (January 2025 to October 2025, *n* = 544) for external validation. A total of 28 candidate variables were collected, covering demographic characteristics, lifestyle and dietary habits, past medical history/medication history/family history, psychological status, and clinical symptoms.

**Results:**

Through multi-dimensional feature selection integrating univariate/multivariate logistic regression, recursive feature elimination (RFE), Boruta algorithm, and clinical expert review, eight key variables were identified. Nine ML models were constructed and optimized, with performance evaluated using metrics including area under the curve (AUC), sensitivity, specificity, and F1-score. The Multilayer Perceptron (MLP) model demonstrated optimal performance, with an area under the receiver operating characteristic curve (AUC) of 0.826 (95% CI: 0.788–0.864) in internal validation and 0.780 (95% CI: 0.745–0.815) in temporal validation. SHAP analysis revealed that Helicobacter pylori infection, age, smoking status, and high-salt pickled food intake were the top risk factors, while fruit and vegetable intake was a protective factor.

**Conclusion:**

This study developed and validated an interpretable ML model for predicting CAG in elderly patients. The model, incorporating eight readily available clinical variables, provides a non-invasive, accessible tool for risk stratification and may facilitate early screening and targeted intervention in primary care settings.

## Introduction

Gastric cancer is the fifth most common malignant tumor and the fifth leading cause of cancer-related deaths worldwide, with a predominantly elderly patient population and a median age at diagnosis of 68 years (1). Despite certain advancements in therapeutic approaches, the prognosis of advanced gastric cancer remains poor, with persistently low 5-year survival rates (2). Therefore, the early and accurate identification of high-risk populations prior to the onset of gastric cancer is crucial for reducing its incidence and mortality.

Chronic atrophic gastritis (CAG) is classified as a precancerous condition that occupies a critical position in the Correa cascade pathway — the well-established stepwise progression from normal gastric mucosa through chronic gastritis, atrophic gastritis, intestinal metaplasia, and dysplasia to gastric cancer (3). The global prevalence of CAG is approximately 30%–35%, and it increases substantially with advancing age, making the elderly population a particularly high-risk group (4). The occurrence of gastric intraepithelial neoplasia is regarded as a precancerous lesion of gastric cancer; therefore, the early identification and standardized management of CAG are fundamental strategies for blocking malignant transformation and reducing the incidence of gastric cancer.

In the current clinical pathway, endoscopy is recognized as the gold standard for CAG screening and diagnosis due to its high diagnostic accuracy. However, the inherent invasiveness of endoscopic procedures causes procedural discomfort and anxiety that significantly reduce compliance among elderly patients. Simultaneously, structural barriers including shortages of endoscopy equipment and trained gastroenterologists in rural and economically disadvantaged areas further limit timely access to diagnostic services (5, 6). These combined factors mean that a large number of potential CAG cases are not detected early, greatly increasing the risk of disease progression to advanced gastric cancer (7, 8).

Machine learning (ML), an important branch of artificial intelligence (AI), has demonstrated advantages in predicting various health outcomes. To address the “black box” nature of ML models, the SHapley Additive exPlanations (SHAP) method provides a unified framework for interpreting ML outputs, enabling the visualization of the individual contributions of each variable in the predictive model (9).

Recent studies have begun applying ML to predict precancerous gastric lesions. Notably, Wang et al. (10) conducted a multicentre cohort study that developed and validated an interpretable ML model using symptom and lifestyle data for non-invasive screening of precancerous gastric lesions, demonstrating the feasibility of ML-based approaches in this domain. However, that study did not specifically target the elderly high-incidence population and omitted comorbidities and psychological factors as candidate predictors, thereby limiting its clinical specificity and applicability to high-risk elderly individuals. To date, research applying ML algorithms specifically for predicting CAG in the elderly while enhancing model interpretability using the SHAP method remains scarce.

Therefore, this study aimed to: (1) develop and compare nine machine learning models (three independent models and six ensemble models) for predicting CAG in elderly patients, and identify the optimal model based on comprehensive evaluation of discriminative ability (AUC), calibration, clinical utility (decision curve analysis), and classification metrics; (2) Conduct rigorous internal and external validation to ensure reliability and accuracy, while using the SHAP method to explain the contribution of predictive factors to chronic atrophic gastritis in the elderly.

## Materials and methods

### Study design

This study adopted a retrospective cohort study (with temporal external validation) design, adhering to the Transparent Reporting of a Multivariable Prediction Model for Individual Prognosis or Diagnosis (TRIPOD) guidelines (11) and the step-by-step guidelines for developing clinical prediction models (12).

This study was approved by the Ethics Committee of Shanghai Tenth People’s Hospital, Tongji University (Approval No.: KLL-2025-476). Due to the retrospective nature of the cohort and the anonymization of data, individual informed consent was waived.

### Study population

Two temporally distinct, non-overlapping cohorts were included: a development cohort (January 1, 2023 to December 31, 2024) for model construction and internal validation, and a temporal validation cohort (January 1, 2025 to October 31, 2025) for external validation. The two cohorts are temporally independent with no patient overlap, ensuring the independence of external validation. Inclusion criteria: ≥ 60 years old, admitted due to abdominal discomfort, completed gastroscopy + biopsy with complete pathological results. Exclusion criteria: pathological results indicating gastric cancer or high-grade intraepithelial neoplasia; previous gastrectomy; severe systemic diseases precluding completion of assessment; unclear pathological results.

### Sample size calculation

Sample size estimation was based on the Events Per Variable (EPV) rule for prediction model development (13), Assuming an EPV of 20 (to ensure model stability), with 8 candidate predictors and an anticipated CAG prevalence of 50%–70%, among elderly patients undergoing endoscopy (4), a minimum of 160 CAG cases (320 total patients) would be required. However, to enhance model robustness and generalizability, we included all consecutive eligible patients during the predefined data collection period (January 2023 to October 2025), resulting in a final sample size of 1,812 patients.

### Definition of chronic atrophic gastritis

CAG was diagnosed based on histopathological examination of gastric mucosal biopsies obtained during endoscopy. The diagnosis required evidence of glandular atrophy, defined as the loss (numerical reduction) of appropriate gastric glands or their replacement by metaplastic glands, according to the updated Sydney System (14). All biopsy specimens were independently reviewed by two experienced gastrointestinal pathologists blinded to clinical information, with disagreements resolved through consensus.

### Data collection

Based on systematic literature review of CAG risk factors, clinical expert consensus, and data availability, 28 candidate predictors were selected and categorized into five domains according to their clinical and etiological relevance: (1) demographic characteristics (7 variables): gender, age, body mass index (BMI), waist circumference, educational level, marital status, and residence (urban/rural); (2) lifestyle and dietary habits (8 variables): smoking status, alcohol consumption, frequency of fresh fruit and vegetable intake, meat/egg/dairy product consumption, pickled food consumption, overheated food intake, spicy food consumption, and regularity of meal times; (3) medical history (7 variables): hypertension, diabetes mellitus, use of non-steroidal anti-inflammatory drugs (NSAIDs), autoimmune diseases, cardiovascular diseases, family history of gastric cancer, and Helicobacter pylori (H. pylori) infection status; (4) psychological status (2 variables): depression and anxiety, assessed using validated screening tools — specifically, the Generalized Anxiety Disorder scale (GAD-7) for anxiety and the Patient Health Questionnaire (PHQ-9) for depression; and (5) gastrointestinal symptoms (4 variables): epigastric pain, bloating, acid reflux, and belching.

The retrospective cohort dataset was manually extracted from the electronic medical record (EMR) system by two members of the research team using standardized Excel spreadsheets, with double verification and cross-checking, and random inspection by a third researcher to ensure accuracy and reliability.

### Data preprocessing

Data cleaning and preprocessing included standardization and conversion of text descriptions into numerical values to ensure the quality and accuracy of the dataset. Continuous variables were retained in their original form. Categorical variables (e.g., gender) were encoded (female = 0, male = 1). Missing value handling: multiple imputation was used to fill in missing data. Patients with overall data missing exceeding 20% (i.e., more than 5 of the 28 candidate variables missing per patient) were excluded.

### Candidate predictor reduction method

Effective feature selection is crucial in predictive modeling, as an excessive number of variables may lead to overfitting and low computational efficiency, while an insufficient number may miss important patterns (15). To construct a high-precision predictive model, this study adopted a multi-dimensional feature selection strategy to optimize the predictor set. Firstly, based on univariate and multivariate Logistic regression analyses, candidate variables with a significance level of *P* < 0.05 were initially screened. Secondly, the recursive feature elimination (RFE) algorithm was introduced to iteratively eliminate the feature with the lowest contribution and retrain the model to determine the optimal size of the feature subset. Furthermore, the Boruta algorithm was used for comprehensive variable importance assessment, ensuring that the selected features have stable predictive capabilities by comparing the significance of real features with shadow features. Finally, the intersection of all initially screened features was subjected to strict review by a team of clinical experts to ensure that while pursuing model statistical performance, all predictive factors with clear clinical significance are retained, thereby enhancing the clinical interpretability and practicality of the model.

### Model construction, evaluation, and statistical analysis

The retrospective cohort data was randomly split once to maintain sample balance, with 70% used for model training and 30% for internal validation. In this study, 9 types of machine learning predictive models were systematically constructed and validated in the Python 3.11 environment, including three independent machine learning models: Logistic Regression (LR), Support Vector Machine (SVM), and Multilayer Perceptron (MLP), as well as six ensemble machine learning models: Random Forest (RF), eXtreme Gradient Boosting (XGBoost), Gradient Boosting Machine (GBM), Adaptive Boosting (AdaBoost), Bagging, and Stacking. The training process of all models uniformly adopted a grid search combined with 10-fold cross-validation strategy for hyperparameter tuning to improve model robustness, reduce overfitting effects, and optimize parameter configuration. The predictive performance of the models was comprehensively and quantitatively evaluated on the internal validation set based on metrics such as AUC, sensitivity, specificity, positive predictive value, negative predictive value, accuracy, and F1-score. The 9 models were systematically ranked based on all performance metrics to determine the optimal model for predicting CAG. External validation used the same metrics to verify the extrapolability of the model.

To interpret the optimal MLP model and explore the contribution of each predictive feature, SHapley Additive exPlanations (SHAP) analysis was conducted. SHAP values are grounded in cooperative game theory’s Shapley value principle and quantify the marginal contribution of each feature to model output, satisfying properties of local accuracy and consistency. For the MLP model, SHAP values were calculated using the KernelSHAP method, which approximates Shapley values via weighted linear regression and is applicable to arbitrary black-box models. SHAP analysis was implemented using the Python SHAP library (version 0.41.0) and visualized through SHAP bar plots, summary plots, and force plots.

All statistical analyses were performed using R software (version 4.4.0, R Foundation for Statistical Computing, Vienna, Austria) and Python (version 3.11) with scikit-learn library for machine learning. Normality of continuous variables was assessed using the Shapiro–Wilk test. Continuous variables following normal distribution were presented as mean ± standard deviation (SD) and compared using independent *t*-tests, while non-normally distributed variables were presented as median and interquartile range (IQR) and compared using Mann–Whitney U tests. Categorical variables were presented as frequencies and percentages, and compared using chi-square tests or Fisher’s exact tests. Missing data were handled using multiple imputation with chained equations (MICE) with 20 iterations and 5 imputed datasets. All statistical tests were two-tailed, with a significance level of (*P* < 0.05).

## Results

### Patient characteristics

**FIGURE 1:**
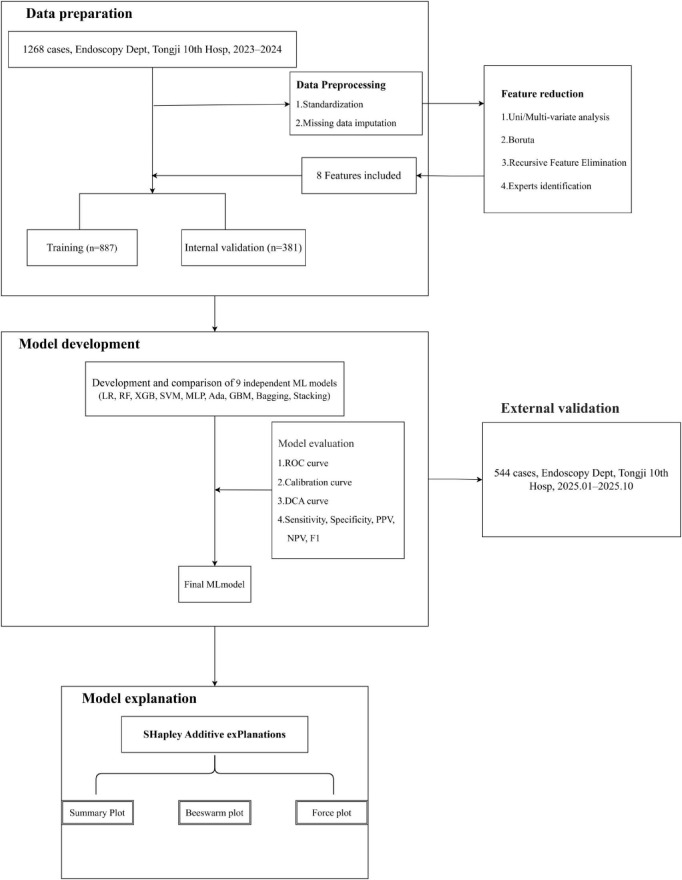
Flow chart.

A total of 1,812 eligible elderly patients were included in the retrospective cohort. Of these, 1,268 patients from the development cohort (January 2023 to December 2024) were used for model construction and internal validation, and 544 patients from the temporally independent validation cohort (January 2025 to October 2025) were used for external validation; the two cohorts are non-overlapping. The demographic and clinical characteristics of the chronic atrophic gastritis group and the non-chronic atrophic gastritis group for model construction are detailed in Table 1, and the demographic and clinical characteristics of the modeling group, internal validation group, and external validation group are shown in Supplementary Table 1. Details of the study design are presented in Figure 1.

**TABLE 1:** Univariate analysis of clinical indicators between chronic atrophic gastritis and non-chronic atrophic gastritis cohorts.

Indicators	Non-chronic atrophic gastritis (*N* = 483)	Chronic atrophic gastritis (*N* = 785)	*t*/z/χ ^2^*	*p*
Age (years)	66 (62, 71)	71 (65, 77)	−8.687z	< 0.001
Waist (cm)	87.73 ± 10.46	88.91 ± 10.53	−1.944t	0.052
BMI (kg/m^2^)	24.61 ± 3.43	24.37 ± 3.21	1.252t	0.211
Gender			0.006χ^2^	0.939
Female	194 (40.17)	317 (40.38)		
Male	289 (59.83)	468 (59.62)		
Education			5.621χ^2^	0.132
College degree or above	110 (22.77)	164 (20.89)		
High school degree	135 (27.95)	207 (26.37)		
Junior high school degree	164 (33.95)	252 (32.1)		
Primary school degree or below	74 (15.32)	162 (20.64)		
Married			0.026χ^2^	0.871
No	156 (32.3)	257 (32.74)		
Yes	327 (67.7)	528 (67.26)		
Residence			6.159χ^2^	0.013
Rural	225 (46.58)	422 (53.76)		
Urban	258 (53.42)	363 (46.24)		
Smoking			81.981χ^2^	< 0.001
No	354 (73.29)	372 (47.39)		
Yes	129 (26.71)	413 (52.61)		
Drinking			4.086χ^2^	0.043
No	310 (64.18)	459 (58.47)		
Yes	173 (35.82)	326 (41.53)		
Fruit and vegetable intake			13.251χ^2^	< 0.001
Low	166 (34.37)	351 (44.71)		
Often	317 (65.63)	434 (55.29)		
Meat, egg, and dairy intake			0.069χ^2^	0.793
Adequate	293 (60.66)	482 (61.4)		
Low	190 (39.34)	303 (38.6)		
High-salt pickled food intake			21.215χ^2^	< 0.001
Frequent	179 (37.06)	395 (50.32)		
No or rare	304 (62.94)	390 (49.68)		
Overheated food intake			16.563χ^2^	< 0.001
Frequent	187 (38.72)	396 (50.45)		
No or rare	296 (61.28)	389 (49.55)		
Spicy food intake			0.190χ^2^	0.663
Frequent	269 (55.69)	447 (56.94)		
No or rare	214 (44.31)	338 (43.06)		
Regular dietary			3.968χ^2^	0.046
Irregular	177 (36.65)	332 (42.29)		
Regular	306 (63.35)	453 (57.71)		
Hypertension			1.745χ^2^	0.186
No	430 (89.03)	679 (86.5)		
Yes	53 (10.97)	106 (13.5)		
Diabetes mellitus			2.062χ^2^	0.151
No	453 (93.79)	719 (91.59)		
Yes	30 (6.21)	66 (8.41)		
NSAIDs			46.988χ^2^	< 0.001
No	434 (89.86)	581 (74.01)		
Yes	49 (10.14)	204 (25.99)		
Autoimmune disease			0.063χ^2^	0.802
No	460 (95.24)	750 (95.54)		
Yes	23 (4.76)	35 (4.46)		
Cardiovascular disease			11.249χ^2^	0.001
No	439 (90.89)	662 (84.33)		
Yes	44 (9.11)	123 (15.67)		
Family history of gastric cancer			4.459χ^2^	0.035
No	465 (96.27)	734 (93.5)		
Yes	18 (3.73)	51 (6.5)		
Helicobacter pylori			102.989χ^2^	< 0.001
Hp-negative	315 (65.22)	282 (35.92)		
Hp-positive	168 (34.78)	503 (64.08)		
Depression			7.328χ^2^	0.007
No depression	443 (91.72)	681 (86.75)		
With depression	40 (8.28)	104 (13.25)		
Anxiety			15.275χ^2^	< 0.001
No anxiety	439 (90.89)	652 (83.06)		
With anxiety	44 (9.11)	133 (16.94)		
Gastric pain			5.315χ^2^	0.021
No gastric pain	390 (80.75)	590 (75.16)		
With gastric pain	93 (19.25)	195 (24.84)		
Bloating			0.114χ^2^	0.735
No bloating	361 (74.74)	580 (73.89)		
With bloating	122 (25.26)	205 (26.11)		
Reflux			2.529χ^2^	0.112
No reflux	376 (77.85)	580 (73.89)		
With reflux	107 (22.15)	205 (26.11)		
Belching			5.889χ^2^	0.015
No belching	388 (80.33)	584 (74.39)		
With belching	95 (19.67)	201 (25.61)		

*t denotes independent samples *t*-test statistic, z denotes Mann–Whitney U test statistic, χ^2^ denotes chi-square test statistic (or Fisher’s exact test). The specific test type is indicated by the superscript symbol appended to each statistic value (e.g., −8.687z = Mann–Whitney U test; 81.981χ^2^ = chi-square test; −1.944t = independent *t*-test). Data types: normally distributed continuous variables are presented as mean ± SD; non-normally distributed continuous variables as median (interquartile range); categorical variables as frequency (percentage).

Between-group comparisons revealed that the CAG group was significantly older than the non-CAG group (median 71 vs. 66 years, *P* < 0.001). In terms of risk factor exposure, the CAG group had markedly higher rates of H. pylori infection (64.08% vs. 34.78%), smoking (52.61% vs. 26.71%), and NSAID use (25.99% vs. 10.14%) compared to the non-CAG group (all *P* < 0.001). Additionally, frequent high-salt pickled food intake (50.32% vs. 37.06%) and frequent overheated food intake (50.45% vs. 38.72%) were significantly more prevalent in the CAG group (both *P* < 0.001). Regarding protective factors, the proportion of frequent fruit and vegetable intake was significantly lower in the CAG group (55.29% vs. 65.63%, *P* < 0.001). Furthermore, the prevalence of anxiety (16.94% vs. 9.11%) and depression (13.25% vs. 8.28%) was significantly higher in the CAG group (both *P* < 0.05).

### Feature selection

Feature selection was performed using a multi-step approach combining statistical and algorithmic methods. Multivariate logistic regression identified 12 variables (*P* < 0.05) (Table 2). The four variables with the largest absolute OR values were highlighted as the most statistically and clinically prominent predictors, each representing a distinct etiological domain: H. pylori infection (OR 4.305, 95% CI 3.248–5.745; infectious domain), NSAID use (OR 3.434, 95% CI 2.341–5.124; iatrogenic domain), smoking (OR 3.291, 95% CI 2.478–4.398; behavioral domain), and anxiety (OR 2.551, 95% CI 1.670–3.962; psychological domain).

**TABLE 2:** Association between clinical variables and risk of chronic atrophic gastritis (logistic regression analysis).

Indicators	Estimate	SE	Statistic	Wald χ 2	OR (95% CI)	*P*
Age (years)	0.094	0.011	8.808	77.581	1.098 (1.076, 1.122)	< 0.001
Residence						
Rural	*R*	0.139	−0.827	0.684	0.891 (0.679, 1.171)	0.408
Urban	−0.115					
Smoking						
No	*R*	0.146	8.145	66.341	3.291 (2.478, 4.398)	< 0.001
Yes	1.191					
Drinking						
No	*R*	0.143	2.039	4.158	1.339 (1.012, 1.775)	0.041
Yes	0.292					
Fruit and vegetable intake						
Low	*R*	0.143	−2.664	7.097	0.683 (0.516, 0.903)	0.008
Often	−0.381					
High-salt pickled food intake						
Frequent	*R*	0.142	−5.300	28.090	0.471 (0.356, 0.621)	< 0.001
No or rare	−0.752					
Overheated food intake						
Frequent	*R*	0.140	−2.964	8.785	0.66 (0.501, 0.868)	0.003
No or rare	−0.415					
Regular dietary						
Irregular	*R*	0.144	−0.722	0.521	0.901 (0.68, 1.195)	0.47
Regular	−0.104					
NSAIDs						
No	*R*	0.200	6.183	38.229	3.434 (2.341, 5.124)	< 0.001
Yes	1.234					
Cardiovascular disease						
No	*R*	0.219	1.699	2.887	1.451 (0.949, 2.244)	0.089
Yes	0.372					
Family history of gastric cancer						
No	*R*	0.331	2.659	7.070	2.408 (1.28, 4.701)	0.008
Yes	0.879					
Helicobacter pylori						
Hp-negative	*R*	0.145	10.039	100.782	4.305 (3.248, 5.745)	< 0.001
Hp-positive	1.46					
Depression						
No depression	*R*	0.236	2.787	7.767	1.928 (1.223, 3.085)	0.005
With depression	0.657					
Anxiety						
No anxiety	*R*	0.220	4.255	18.105	2.551 (1.67, 3.962)	< 0.001
With anxiety	0.937					
Gastric pain						
No gastric pain	*R*	0.172	1.663	2.766	1.33 (0.952, 1.867)	0.096
With gastric pain	0.286					
Belching						
No belching	*R*	0.168	2.245	5.040	1.458 (1.051, 2.033)	0.025
With belching	0.377					

**FIGURE 2:**
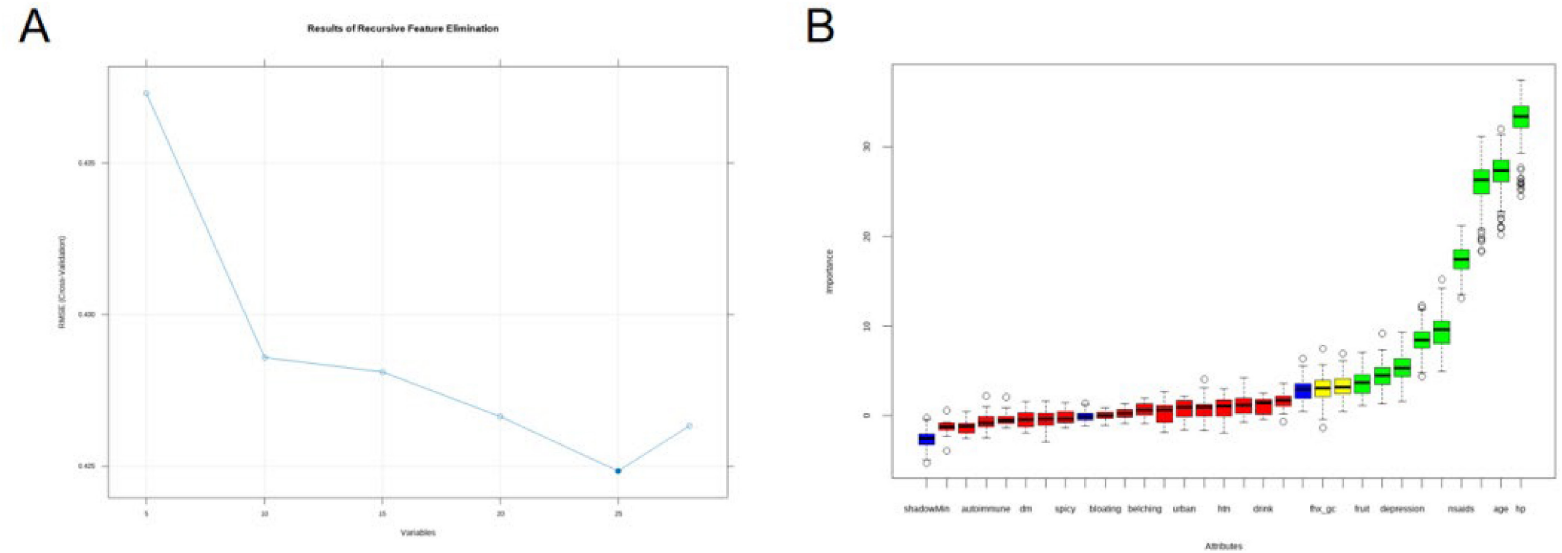
Variable selection. **(A)** Results of recursive feature elimination (RFE) algorithm, showing the change in model performance with the progressive reduction of feature dimensions. **(B)** Forest plot displaying the feature importance ranking and corresponding effect sizes derived from the final predictive model.

The recursive feature elimination (RFE) algorithm identified an optimal feature subset of 25 variables (Figure 2A), while the Boruta algorithm confirmed 9 variables as having consistently confirmed importance (Figure 2B). The intersection of these three methods (multivariate logistic regression, RFE, and Boruta) yielded 8 overlapping variables: age, smoking status, fruit and vegetable intake, high-salt pickled food intake, NSAID use, H. pylori infection, depression, and anxiety. Overheated food intake, which appeared in the multivariate regression results, exhibited clinical collinearity with high-salt pickled food intake and was removed following clinical expert review, with the clinically more etiologically specific variable (high-salt pickled food intake) retained. This intersection approach, combined with clinical expert review to ensure biological plausibility and clinical interpretability, yielded the final set of eight predictors.

### Model construction

Based on the above-selected predictive variables, 9 machine learning models (LR: Logistic Regression, RF: Random Forest, XGB: eXtreme Gradient Boosting, SVM: Support Vector Machine, MLP: Multilayer Perceptron, GBM: Gradient Boosting Machine, Ada: Adaptive Boosting, Bagging, Stack: Stacking) were constructed on the training set, and 10-fold cross-validation was used for hyperparameter selection. [Model construction methodology is described in detail in the Methods section.] The optimal parameters are shown in Supplementary Table 2.

### Model evaluation

**FIGURE 3:**
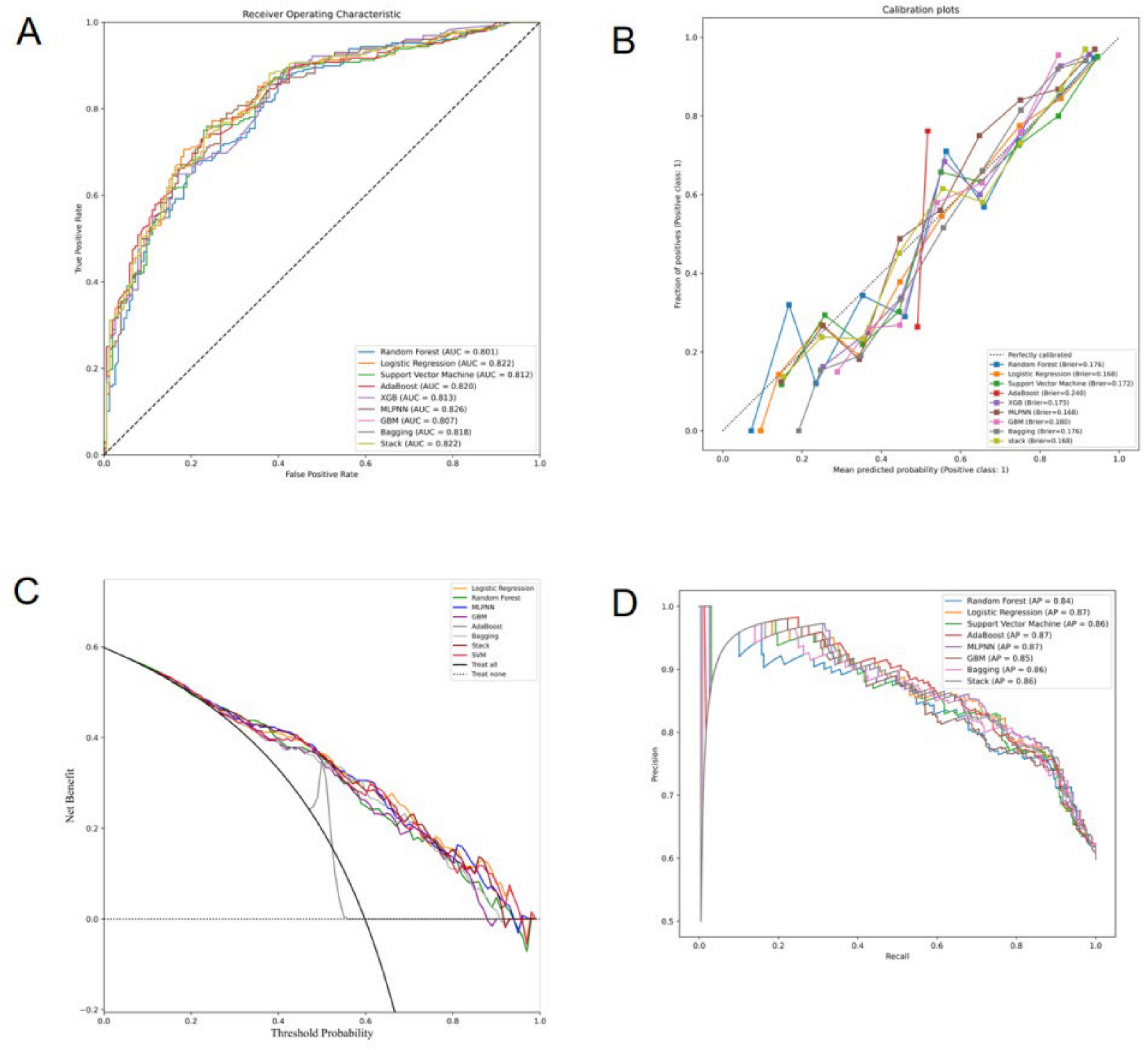
Performance evaluation of machine learning models. **(A)** Receiver operating characteristic (ROC) curves; **(B)** calibration curves; **(C)**: decision curve analysis (DCA); **(D)**: precision-recall curves.

The predictive performance of nine machine learning models was comprehensively evaluated using multiple metrics (Table 3 and Figure 3). In the internal validation set, the MLP model achieved the highest AUC of 0.826 (95% CI 0.788–0.864). The MLP model also demonstrated well-balanced sensitivity (0.750) and specificity (0.752), avoiding the common trade-off between these metrics observed in other models. Calibration analysis showed that the MLP model’s predicted probabilities closely aligned with observed event rates, indicating excellent calibration (Figure 3B). Decision curve analysis revealed that the MLP model provided superior net benefit across a wide range of threshold probabilities (0.2–0.8), outperforming both the treat-all and treat-none strategies (Figure 3C). The precision-recall curve confirmed robust performance with an average precision of 0.843 (Figure 3D). Based on these comprehensive evaluations, the MLP model was selected as the optimal model for subsequent external validation and clinical application.

**TABLE 3:** Performance metrics of different machine learning models.

Model	LR	RF	XGB	SVM	MLP	GBM	Ada	Bagging	Stack
AUC	0.822	0.801	0.813	0.812	0.826	0.807	0.82	0.818	0.822
Specificity	0.758	0.712	0.693	0.758	0.752	0.673	0.732	0.771	0.739
Sensitivity	0.754	0.719	0.724	0.759	0.75	0.759	0.741	0.689	0.763
NPV	0.674	0.63	0.627	0.678	0.669	0.652	0.655	0.624	0.677
PPV	0.823	0.788	0.778	0.824	0.818	0.776	0.805	0.818	0.813

### External validation of the optimal model

The optimal MLP model was validated in an independent temporal cohort of 544 patients. The model maintained good discriminative ability with an AUC of 0.780 (95% CI 0.745–0.815), representing a modest decrease from the internal validation AUC of 0.826. The sensitivity decreased from 0.750 to 0.672, while specificity remained relatively stable (0.752 vs. 0.787). The modest performance decrease in temporal validation may reflect temporal changes in patient characteristics or clinical practice patterns between the development and validation periods.

### SHAP visualization

**FIGURE 4:**
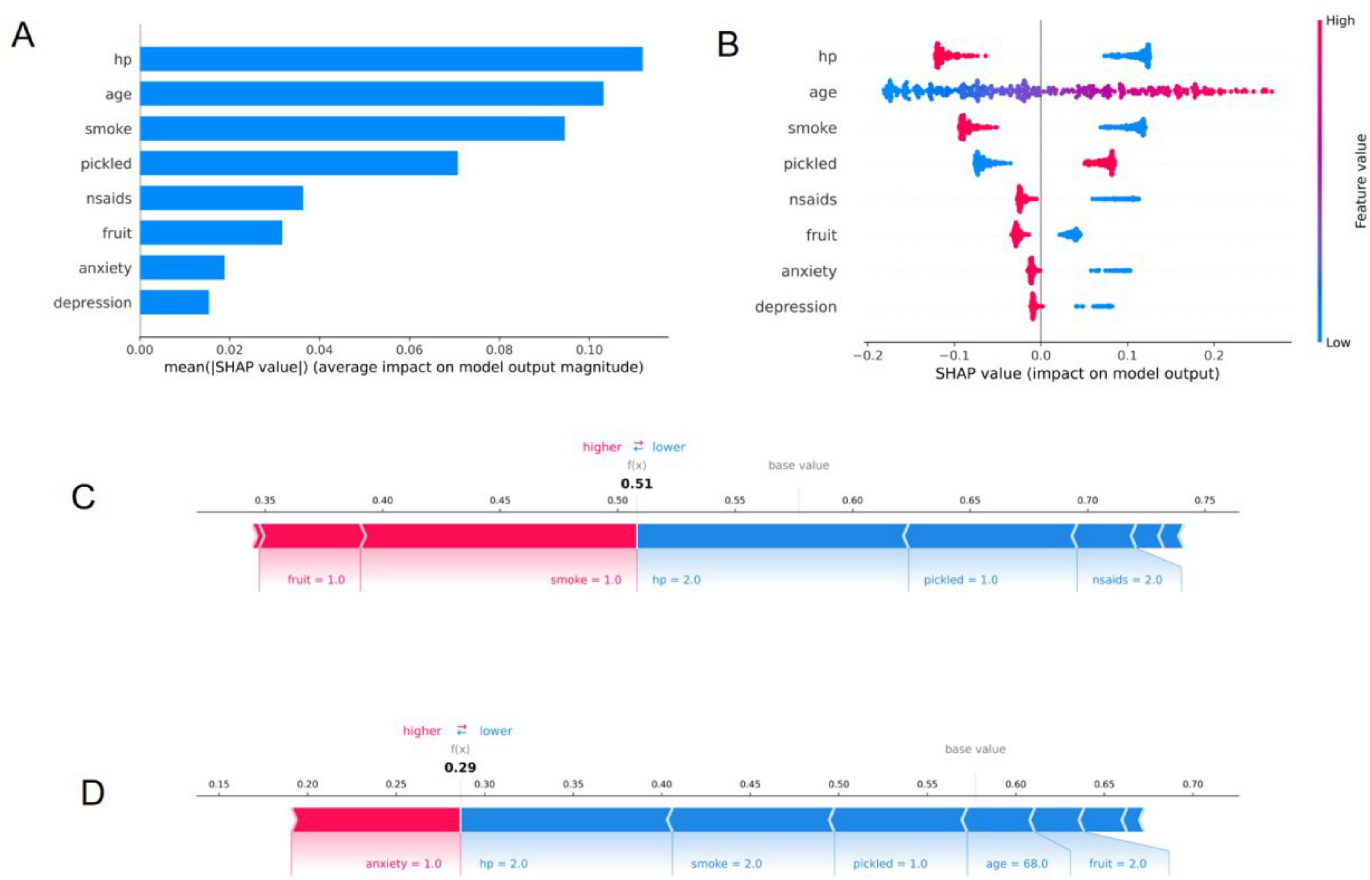
SHAP-based feature importance and impact analysis of the optimal MLP model. **(A)** Mean absolute SHAP values of predictive features (ranked by importance); **(B)** SHAP summary plot of feature impact on model output; **(C)** SHAP force plot for a representative high-risk sample; **(D)** SHAP force plot for a representative low-risk sample.

To interpret the optimal MLP model and explore the contribution of each predictive feature, we conducted SHapley Additive exPlanations (SHAP) analysis (Figure 4).

Figure 4A shows the mean absolute SHAP values, ranking features by their overall impact on model output. Helicobacter pylori (Hp) infection had the strongest influence, followed by age, smoking status, and high-salt pickled food intake, indicating these were the most critical drivers of risk prediction. Figure 4B presents the SHAP summary plot, where the color gradient (red = high feature value, blue = low feature value) and SHAP value direction illustrate how each feature affects the model’s risk prediction. For example, a positive Hp status (red) increased the predicted risk (positive SHAP value), while higher fruit intake (blue) reduced the risk (negative SHAP value). Similarly, current smoking (red) elevated risk, whereas regular fruit consumption (blue) mitigated it. Figure 4C displays the SHAP force plot for a representative high-risk sample, with a baseline risk of 0.51. Red features (e.g., smoking, low fruit intake) pushed the risk upward, while blue features (e.g., Hp-positive status, high-salt pickled food intake) further increased the cumulative risk, resulting in a significantly elevated final prediction. Figure 4D shows the SHAP force plot for a representative low-risk sample, with a baseline risk of 0.29. Although red features (e.g., anxiety) slightly increased risk, blue features (e.g., Hp-negative status, high fruit intake, younger age) collectively pulled the risk downward, maintaining a low final prediction.

## Discussion

To the best of our knowledge, this study is the first to systematically apply machine learning algorithms to identify predictive risk indicators, develop 9 machine learning models, evaluate their performance, and select the optimal model for predicting the risk of chronic atrophic gastritis in the elderly. The results of the study indicate that based on the comprehensive performance ranking, the MLP model is the optimal model. While previous studies have explored machine learning approaches for predicting gastric precancerous lesions (11), our study advances the field in several important ways. First, we specifically targeted the elderly population (≥ 60 years), who bear the highest CAG burden but are often underrepresented in predictive modeling studies. Second, we incorporated a comprehensive set of 28 candidate variables spanning demographic, lifestyle, dietary, medical, psychological, and symptomatic domains, whereas prior studies primarily focused on laboratory biomarkers or endoscopic features (11). Third, we systematically compared nine machine learning algorithms using rigorous multi-dimensional evaluation (discriminative ability, calibration, clinical utility, and classification performance) rather than relying solely on AUC. Fourth, we employed SHAP analysis to provide transparent, clinically interpretable explanations of individual risk predictions, addressing the “black box” criticism commonly leveled at machine learning models. Finally, we demonstrated model generalizability through temporal validation in an independent cohort, which is critical for assessing real-world applicability.

Feature selection is crucial in the development of machine learning models, directly affecting predictive accuracy and clinical applicability. However, there is currently a lack of standardized guidelines for determining the optimal number or type of features in predictive models (16, 17). Variable screening methods based on influencing factor analysis mostly select variables with *P* < 0.05 for inclusion in the model, which may miss important variables or introduce other noisy variables (18). The RFE algorithm may select feature subsets that perform well on the training set but have poor generalization ability (19), and the Boruta algorithm may overfit in high-dimensional datasets (20). Taking the intersection of the three feature selection methods and confirming with clinical experts can maximize the avoidance of missing effective predictive variables and enhance the extrapolability of the model. The final selection of eight predictors through our multi-method approach (univariate/multivariate regression, RFE, Boruta, and clinical review) represents a balance between model parsimony and predictive accuracy. These eight variables (H. pylori infection, age, smoking, fruit/vegetable intake, pickled food intake, NSAID use, depression, and anxiety) are readily obtainable in primary care settings without requiring invasive procedures or costly laboratory tests, enhancing the model’s practical applicability. Notably, some variables that showed statistical significance in univariate analysis (e.g., residence, cardiovascular disease) were not retained in the final model. This decision was based on their marginal contribution in multivariate models and potential confounding with other variables (e.g., residence may correlate with dietary patterns). The exclusion of laboratory biomarkers (e.g., pepsinogen, gastrin-17) distinguishes our model from others (11) but was intentional to maximize accessibility in resource-limited settings. While biomarker-based models may achieve higher discrimination, our clinical variable-based approach offers superior feasibility for population-level screening, particularly in primary care or community settings where laboratory testing may not be readily available.

To ensure the robustness of the study conclusions, this study compared the predictive performance of 9 mainstream machine learning models (Logistic Regression, Random Forest, eXtreme Gradient Boosting, Support Vector Machine, Multilayer Perceptron, etc.) using core evaluation metrics including AUC, sensitivity, specificity, positive predictive value, and negative predictive value. The results showed that the Multilayer Perceptron (MLP) model performed the best (AUC = 0.826), demonstrating overall superior performance in terms of discriminative ability, calibration, and clinical net benefit. It should be noted that the AUC difference between MLP and the next-best model (LR, AUC = 0.822) was modest, and the final model selection additionally considered calibration performance and clinical net benefit as multi-dimensional criteria. Compared with traditional statistical methods, machine learning models can more efficiently handle multi-factor interaction effects and avoid the limitations of linear assumptions, making them particularly suitable for risk factor mining of complex multi-etiology diseases such as CAG. This is also an important reason why this study can accurately identify the synergistic effects of dietary, psychological, and physiological factors.

Furthermore, the interpretable decomposition of the optimal MLP model through SHAP analysis clarified the impact strength and direction of each factor on CAG risk, addressing the “black box” problem of traditional machine learning models. The SHAP feature importance ranking showed that Hp infection had the strongest impact on the model output, followed by age, smoking, and high-salt pickled food intake, while fruit and vegetable intake played an antagonistic role as core protective factors. This ranking is highly consistent with clinical cognition and existing research conclusions, enhancing the clinical persuasiveness of the model results. At the same time, the SHAP summary plot and force plot clearly revealed the dose-response relationship of each factor. For example, Hp positivity and long-term smoking can significantly increase CAG risk, while adequate fruit and vegetable intake can partially offset the adverse effects of high-risk factors, providing specific evidence for the clinical application of dietary intervention.

This study found that age, smoking, Hp infection, long-term use of NSAIDs, high-salt pickled food dietary pattern, depression, and anxiety are significant high-risk factors for chronic atrophic gastritis (CAG), which is consistent with previous research results, further verifying the core role of these factors in the occurrence and development of CAG, and providing evidence-based basis for the risk stratification and prevention of CAG.

As the most influential risk factor in this study, Hp infection is highly consistent with the conclusions of multiple epidemiological studies (21, 22). Hp colonizes gastric mucosal epithelial cells, releases pathogenic factors such as urease and vacuolating toxin, destroys the integrity of the gastric mucosal barrier, induces chronic inflammatory responses, and long-term stimulation can lead to atrophy of intrinsic gastric glands, and even progress to intestinal metaplasia and dysplasia. Previous studies have confirmed that eradicating Hp can significantly delay the process of gastric mucosal atrophy (23), which also provides a mechanistic basis for the strong association between Hp infection and CAG in this study. Active Hp screening and standardized eradication treatment can help reduce, to some extent, the risk of CAG and its progression to gastric cancer (24). The results of this study further strengthen the important position of Hp screening and eradication in the primary prevention of CAG, especially for high-risk populations, Hp detection and standardized treatment should be prioritized.

As an uncontrollable risk factor, the association between age and CAG is consistent with the physiological aging law and pathological cumulative effect of gastric mucosa (25). With increasing age, gastric mucosal blood flow decreases, glands naturally atrophy, secretory function declines, mucosal repair ability weakens, and the cumulative effect of long-term exposure to various environmental risk factors (such as diet, smoking) increases significantly, leading to a gradual increase in the risk of CAG with age. This suggests that clinical practice should strengthen the monitoring of gastric mucosal health in the elderly, conduct regular endoscopic screening, and achieve early detection and intervention of CAG (26).

The significant association between smoking and CAG was confirmed in this study, and its pathogenic effect is dose-dependent (27). Harmful substances such as nicotine and tar in tobacco can act on the gastric mucosa through the blood circulation, inhibit the synthesis of gastric mucosal prostaglandins, reduce gastric mucus secretion, aggravate gastric mucosal ischemia and hypoxia, and promote gastric acid secretion, further damaging the gastric mucosal barrier, inducing chronic inflammation, and accelerating glandular atrophy. This suggests that smoking cessation should be an important intervention measure for CAG prevention and control. Long-term use of NSAIDs is an important iatrogenic risk factor for CAG, and its pathogenic mechanism is mainly related to the inhibition of cyclooxygenase (COX) activity (28). COX-1-mediated prostaglandins are key substances for maintaining the integrity of the gastric mucosal barrier. Long-term use of NSAIDs can inhibit COX-1 activity, reduce prostaglandin synthesis, lead to a decrease in gastric mucosal defense capacity, and directly stimulate gastric mucosal epithelial cells, inducing inflammatory responses. Long-term use can lead to gastric mucosal erosion, ulcers, and then develop into atrophy. The results of this study are consistent with the conclusions of the guidelines of the International Society of Gastroenterology, which clearly recommend that populations with long-term use of NSAIDs should take gastric mucosal protectants simultaneously and conduct regular gastric mucosal assessments to reduce the risk of CAG and peptic ulcers (29). As an important dietary risk factor, the high-salt pickled food dietary pattern is closely related to high-salt stimulation and nitrite exposure. High-salt diet can directly stimulate the gastric mucosa, leading to mucosal hyperemia and edema, promoting the release of inflammatory factors, and can induce glandular atrophy in the long term; at the same time, nitrite contained in pickled food can be converted into nitrosamine in the acidic environment of the stomach. Nitrosamine, as a clear pre-carcinogenic substance, can aggravate gastric mucosal damage and accelerate CAG progression (30). The results of this study are consistent with studies in East Asian countries such as Japan and South Korea.

The significant association between depression, anxiety, and CAG reveals the important significance of the “mind-body interaction” in gastric mucosal diseases (31). Depression and anxiety can affect gastrointestinal motility and secretory function through the hypothalamic-pituitary-adrenal (HPA) axis and autonomic nervous system dysfunction, leading to abnormal gastric acid secretion, reduced gastric mucosal blood flow, and simultaneously inhibiting immune function, aggravating gastric mucosal inflammatory responses, delaying mucosal repair, and thereby promoting the occurrence and development of CAG.

This study found that fruit and vegetable intake is a protective factor for chronic atrophic gastritis. Previous studies have confirmed that daily intake of sufficient fresh fruits and vegetables can significantly reduce the risk of CAG, and the frequency of intake is positively correlated with the protective effect (32). Especially in high-risk populations such as Hp infection and high-salt diet, adequate fruit and vegetable intake can partially offset the adverse effects of risk factors, reflecting the feasibility and importance of dietary intervention in CAG prevention and control (33).

The external validation AUC of 0.780 represents a modest decrease from the internal validation AUC of 0.826 (ΔAUC = 0.046), which remains within a clinically acceptable range. To assess whether this decline reflects overfitting, we compared the AUC trajectory across the training set (AUC ≈ 0.826), internal validation set (AUC = 0.826), and external temporal validation set (AUC = 0.780). The close agreement between training and internal validation performance indicates an absence of the typical overfitting pattern (where training performance substantially exceeds validation performance). Therefore, the performance decline in external validation is more likely attributable to distributional shifts in key risk factor exposures between the two cohorts — such as differences in fruit and vegetable intake habits and high-salt pickled food consumption prevalence — as well as temporal changes in patient characteristics or clinical practice patterns occurring across the 2023–2025 study period, rather than model overfitting.

Several limitations should be acknowledged. First, the single-center retrospective design may limit generalizability to other geographic regions and healthcare settings. Multi-center prospective validation, particularly in diverse populations, is needed to confirm model performance. Second, dietary assessment relied on categorical classifications (frequent vs. infrequent intake) rather than quantitative measures, precluding dose-response analysis. Future studies incorporating detailed dietary records could refine these associations. Third, we did not include serological biomarkers (e.g., pepsinogen I/II ratio, gastrin-17) in our model; while this enhances accessibility, it may limit discriminative performance compared to biomarker-integrated models. Fourth, the cross-sectional design precludes causal inference; prospective cohort studies are needed to definitively establish temporal relationships between risk factors and CAG development. Fifth, we could not adjust for all potential confounders (e.g., H. pylori strain virulence, detailed medication history), which may have influenced observed associations. Sixth, future studies should explore SHAP interaction plots for anxiety and depression to reveal the impact of comorbid psychological distress on CAG risk, and calculate the Net Reclassification Index (NRI) to quantify the incremental predictive value added by psychological factors over a baseline physiological-lifestyle model. Finally, the model has not yet been implemented in clinical practice; prospective implementation studies are needed to evaluate real-world effectiveness, acceptability, and impact on screening uptake and clinical outcomes.

## Conclusion

In conclusion, we developed an interpretable machine learning model to implement precise screening and stratified intervention for high-risk populations, such as strengthening Hp eradication, smoking cessation and alcohol restriction, increasing fresh fruit and vegetable intake, reducing high-salt pickled food consumption, standardizing NSAIDs use, and psychological counseling, so as to reduce the incidence of CAG and delay disease progression. In the future, prospective studies are needed to further explore the interaction and mechanisms of various factors, clarify the differences in the protective effects of different fruits and vegetables, and provide more comprehensive evidence-based support for the precise prevention and control of CAG.

## Data Availability

The original contributions presented in this study are included in the article/Supplementary material, further inquiries can be directed to the corresponding author.
