# Supplementary material for: Development and validation of an interpretable machine learning model for predicting chronic atrophic gastritis in elderly patients
Source: Front Med (Lausanne). 2026 Jul 2;13:1810290. doi: 10.3389/fmed.2026.1810290 (PMC13372595; doi:10.3389/fmed.2026.1810290)
Supplement: Supplementary file 1 [file Table_1.docx]

**Table S1. Comparison of Demographic and Clinical Indicators Among the Training, Internal Validation, and External Validation Sets**

| **Indicators** | **Training set** | **Internal validation** | | **External validation** | **t/z/χ^2^*** | ***p*** |
| --- | --- | --- | --- | --- | --- | --- |
| **Gender** |  |  |  | | 1.728 | 0.422 |
| Female | 349(39.35) | 162(42.52) | 230(42.28) | |  |  |
| Male | 538(60.65) | 219(57.48) | 314(57.72) | |  |  |
| **Education** |  |  |  | | 4.592 | 0.597 |
| College degree or above | 197(22.21) | 77(20.21) | 122(22.43) | |  |  |
| High school degree | 238(26.83) | 104(27.3) | 156(28.68) | |  |  |
| Junior high school degree | 298(33.6) | 118(30.97) | 170(31.25) | |  |  |
| Primary school degree or below | 154(17.36) | 82(21.52) | 96(17.65) | |  |  |
| **Married** |  |  |  | | 0.148 | 0.929 |
| No | 286(32.24) | 127(33.33) | 178(32.72) | |  |  |
| Yes | 601(67.76) | 254(66.67) | 366(67.28) | |  |  |
| **Residence** |  |  |  | | 1.338 | 0.512 |
| Rural | 445(50.17) | 202(53.02) | 268(49.26) | |  |  |
| Urban | 442(49.83) | 179(46.98) | 276(50.74) | |  |  |
| **Smoking** |  |  |  | | 2.207 | 0.332 |
| No | 514(57.95) | 212(55.64) | 329(60.48) | |  |  |
| Yes | 373(42.05) | 169(44.36) | 215(39.52) | |  |  |
| **Drinking** |  |  |  | | 1.546 | 0.462 |
| No | 530(59.75) | 239(62.73) | 340(62.5) | |  |  |
| Yes | 357(40.25) | 142(37.27) | 204(37.5) | |  |  |
| **Fruit and vegetable intake** |  |  |  | | 5.81 | 0.055 |
| Low | 357(40.25) | 160(41.99) | 190(34.93) | |  |  |
| Often | 530(59.75) | 221(58.01) | 354(65.07) | |  |  |
| **Meat, egg, and dairy intake** |  |  |  | | 3.666 | 0.16 |
| Adequate | 554(62.46) | 221(58.01) | 316(58.09) | |  |  |
| Low | 333(37.54) | 160(41.99) | 228(41.91) | |  |  |
| **High-salt pickled food intake** |  |  |  | | 0.914 | 0.633 |
| Frequent | 394(44.42) | 180(47.24) | 243(44.67) | |  |  |
| No or rare | 493(55.58) | 201(52.76) | 301(55.33) | |  |  |
| **Overheated food intake** |  |  |  | | 1.571 | 0.456 |
| Frequent | 418(47.13) | 165(43.31) | 249(45.77) | |  |  |
| No or rare | 469(52.87) | 216(56.69) | 295(54.23) | |  |  |
| **Spicy food intake** |  |  |  | | 1.886 | 0.389 |
| Frequent | 495(55.81) | 221(58.01) | 291(53.49) | |  |  |
| No or rare | 392(44.19) | 160(41.99) | 253(46.51) | |  |  |
| **Regular dietary** |  |  |  | | 2.675 | 0.263 |
| Irregular | 343(38.67) | 166(43.57) | 217(39.89) | |  |  |
| Regular | 544(61.33) | 215(56.43) | 327(60.11) | |  |  |
| **Hypertension** |  |  |  | | 4.626 | 0.099 |
| No | 767(86.47) | 342(89.76) | 462(84.93) | |  |  |
| Yes | 120(13.53) | 39(10.24) | 82(15.07) | |  |  |
| **Diabetes mellitus** |  |  |  | | 0.974 | 0.614 |
| No | 824(92.9) | 348(91.34) | 501(92.1) | |  |  |
| Yes | 63(7.1) | 33(8.66) | 43(7.9) | |  |  |
| **NSAIDs** |  |  |  | | 0.817 | 0.665 |
| No | 707(79.71) | 308(80.84) | 444(81.62) | |  |  |
| Yes | 180(20.29) | 73(19.16) | 100(18.38) | |  |  |
| **Autoimmune disease** |  |  |  | | 5.097 | 0.078 |
| No | 852(96.05) | 358(93.96) | 509(93.57) | |  |  |
| Yes | 35(3.95) | 23(6.04) | 35(6.43) | |  |  |
| **Cardiovascular disease** |  |  |  | | 0.596 | 0.742 |
| No | 768(86.58) | 333(87.4) | 466(85.66) | |  |  |
| Yes | 119(13.42) | 48(12.6) | 78(14.34) | |  |  |
| **Family history of gastric cancer** |  |  |  | | 0.724 | 0.696 |
| No | 841(94.81) | 358(93.96) | 518(95.22) | |  |  |
| Yes | 46(5.19) | 23(6.04) | 26(4.78) | |  |  |
| **Helicobacter pylori** |  |  |  | | 5.327 | 0.07 |
| Hp-negative | 405(45.66) | 192(50.39) | 280(51.47) | |  |  |
| Hp-positive | 482(54.34) | 189(49.61) | 264(48.53) | |  |  |
| **Depression** |  |  |  | | 0.234 | 0.89 |
| No depression | 784(88.39) | 340(89.24) | 484(88.97) | |  |  |
| With depression | 103(11.61) | 41(10.76) | 60(11.03) | |  |  |
| **Anxiety** |  |  |  | | 5.116 | 0.077 |
| No anxiety | 751(84.67) | 340(89.24) | 474(87.13) | |  |  |
| With anxiety | 136(15.33) | 41(10.76) | 70(12.87) | |  |  |
| **Gastric pain** |  |  |  | | 1.362 | 0.506 |
| No gastric pain | 689(77.68) | 291(76.38) | 408(75) | |  |  |
| With gastric pain | 198(22.32) | 90(23.62) | 136(25) | |  |  |
| **Bloating** |  |  |  | | 1.034 | 0.596 |
| No bloating | 651(73.39) | 290(76.12) | 403(74.08) | |  |  |
| With bloating | 236(26.61) | 91(23.88) | 141(25.92) | |  |  |
| **Reflux** |  |  |  | | 1.785 | 0.41 |
| No reflux | 672(75.76) | 284(74.54) | 425(78.12) | |  |  |
| With reflux | 215(24.24) | 97(25.46) | 119(21.88) | |  |  |
| **Belching** |  |  |  | | 0.732 | 0.694 |
| No belching | 681(76.78) | 291(76.38) | 407(74.82) | |  |  |
| With belching | 206(23.22) | 90(23.62) | 137(25.18) | |  |  |
| **Age (years)** | 68(63,75) | 69(64,75) | 68.5(63,76) | | 0.621 | 0.733 |
| **Waist(cm)** | 88.83±10.62 | 87.61±10.22 | 88.2±10.2 | | 1.943 | 0.144 |
| **BMI (kg/m²)** | 24.56 ± 3.34 | 24.25 ± 3.18 | 24.47 ± 3.08 | | 1.162 | 0.313 |

Note: t denotes the test statistic for the independent-samples t test, z denotes the test statistic for the Mann–Whitney U test, and χ² denotes the test statistic for the chi-square test or Fisher’s exact test. †Waist circumference and BMI data were verified and corrected; the two rows had been inadvertently interchanged in the original manuscript and have now been corrected. Data are presented as mean ± standard deviation for normally distributed continuous variables, median (interquartile range) for non-normally distributed continuous variables, and frequency (percentage) for categorical variables.

**Table S2. Hyperparameter Settings of the Machine Learning Models**

| **No.** | **Model** | **Hyperparameters** |
| --- | --- | --- |
| 1 | RF | {'max depth': 10, 'min samples leaf': 4, 'min samples split': 2, 'n estimators': 100} |
| 2 | LR | {'C': 1, 'penalty': 'l1'} |
| 3 | SVM | {'gamma': 0.1, 'kernel': 'linear'} |
| 4 | Ada | {'learning rate': 0.1, 'n estimators': 200} |
| 5 | MLP | {'activation': 'relu', 'alpha': 0.001, 'hidden layer sizes': (100,), 'solver': 'adam'} |
| 6 | GBM | {'learning rate': 0.01, 'max depth': 4, 'min samples leaf': 3, 'min samples split': 2, 'n estimators': 150, 'subsample': 0.8} |
| 7 | XGB | {'learning rate': 0.01, 'max depth': 3, 'n estimators': 300} |
| 8 | Bagging | {'max features': 0.9, 'max samples': 0.3, 'n estimators': 50} |
| 9 | Stacking | {'final estimator C': 10, 'stack method': 'auto'} |
